# Identifying top 10 primary care research priorities from international stakeholders using a modified Delphi method

**DOI:** 10.1371/journal.pone.0206096

**Published:** 2018-10-25

**Authors:** Braden O’Neill, Vanessa Aversa, Katherine Rouleau, Kim Lazare, Frank Sullivan, Nav Persaud

**Affiliations:** 1 Department of Family and Community Medicine, North York General Hospital, Toronto, Ontario, Canada; 2 Department of Family and Community Medicine, Faculty of Medicine, University of Toronto, Toronto, Ontario, Canada; 3 Undergraduate Medical Education, Faculty of Medicine, University of Toronto, Toronto, Ontario, Canada; 4 Department of Family and Community Medicine, St. Michael’s Hospital, Toronto, Ontario, Canada; 5 School of Medicine, University of St. Andrews, St. Andrews, United Kingdom; 6 Centre for Urban Health Solutions, St. Michael’s Hospital, Toronto, Ontario, Canada; Universitat Bremen, GERMANY

## Abstract

**Background:**

High quality primary care is fundamental to achieving health for all. Research priority setting is a key facilitator of improving how research activity responds to concrete needs. There has never before been an attempt to identify international primary care research priorities, in order to guide resource allocation and to enhance global primary care. This study aimed to identify a list of top 10 primary care research priorities, as identified by members of the public, health professionals working in primary care, researchers, and policymakers.

**Methods:**

We adapted the James Lind Alliance Priority Setting Partnership process, to conduct multiple rounds of stakeholder recruitment and prioritization. The study included an online survey conducted in three languages, followed by an in-person priority setting exercise involving primary care stakeholders from 13 countries.

**Findings:**

Participants identified a list of top 10 international primary care research priorities. These were focused on diverse topics such as enhancing use of information and communication technology, and improving integration of indigenous communities’ knowledge in the design of primary care services. The main limitations of the study related to challenges in engaging an adequate diversity and number of appropriate stakeholders, particularly members of the public, in aggregating the diverse set of responses into coherent categories representative of the participants’ perspectives and in adequately representing the diversity of submitted responses while ensuring research priorities on the final list are sufficiently actionable to guide resource allocation.

**Conclusions:**

The top 10 identified research priorities have the potential to guide research resource allocation, supporting funding agencies and initiatives to promote global primary care research and practice.

## Introduction

Strong primary care is the cornerstone of high-functioning health systems [1,2]. Like any other area of medicine, primary care requires high quality research for optimal performance [3,4]. Primary care is chronically underfunded throughout the world; efforts to advocate for increases in funding to 15% of total health system expenditures have been met with mixed success [5].

The clinical research agenda and generation of research priorities has traditionally been driven by researchers and funding bodies, rather than patients, caregivers, and clinicians [6,7]. As a result, medical research is not always patient-centered and can lack applicability in the clinical setting [8]. Recent evidence encourages patient, caregiver, and clinician engagement in the research process as stakeholders rather than simply as participants, in order ensure current research directions are relevant to patients [7,9]. By gathering patient feedback about the research agenda and the research process, funding bodies and decision-makers could improve resource allocation and discovery in areas of patient need [6,10]. Given the value of primary care to health systems, and recent efforts towards primary care strengthening in low- and middle- income countries (LMIC) such as the Primary Health Care Performance Initiative (PHPCI) [11], it is important to establish research priorities in order to guide investment and resource allocation. Setting research priorities could serve to not only promote research in areas of patient importance, but also to reduce unnecessary research initiatives that do not use or sufficiently improve existing evidence [12]. Thus, establishing priorities to guide future research directions in primary care would help to increase the effectiveness of research funding and decrease research waste.

Although several studies have incorporated patient engagement in research priority setting, such as those affiliated with the James Lind Alliance, they have not reflected global interests but rather cater to the interests of a subset of the population, such as those with a specific disease or those undergoing a specific treatment [9,10,13,14]. This approach supports an increasing emphasis on ‘horizontal’ (i.e. system-wide) efforts in primary care strengthening, rather than ‘vertical’ (i.e. disease-specific) approaches, which although effective for reducing morbidity and mortality from specific conditions, have been criticized for detrimentally affecting local primary care workforce and resources [15].

While primary care research priorities have been suggested in individual reports, a comprehensive, patient-centered study of research priorities in primary care has not yet been conducted [16–18]. Therefore, establishment of key international research priorities in primary care would encourage contribution to this growing field of research, and strengthen future evidence in primary care to improve health outcomes. This study aimed to identify a set of international primary care research priorities to guide resource allocation and research agendas globally it focused on identifying research priorities for family medicine, to support patient oriented research with a focus on LMIC settings.

## Methods

This project was reviewed and approved by the North York General Hospital Research Ethics Board in Toronto, Ontario, Canada (study approval: #16–0046). Consent was considered implied by participation in the study, as approved by the Research Ethics Board. The design, methods, and analysis of this project are modeled primarily on the James Lind Alliance (JLA) process. The JLA method is an inclusive and validated priority-setting method that integrates multiple means of data collection, such as surveys and focus groups [13]. These methods can be feasibly adapted for an international study to provide opportunities for input from a variety of stakeholders, thereby ensuring that diverse perspectives are reflected. The JLA priority-setting process (PSP) was initially developed to focus on an individual condition to identify uncertainties about the effects of treatments or aspects related to disease management or prognosis. We adapted this methodology to focus on primary care more broadly, since one of the key issues identified around a lack of sustainable investment in primary care is an emphasis on “vertical” (that is, disease-specific) priorities as opposed to “horizontal” (system-wide) priorities [11].

The JLA approach is based on the Delphi process, which is an established process for consensus development among stakeholders [19]. The Delphi process typically involves several rounds of item submission and prioritization through the use of structured questionnaires [20]. In the JLA approach, priorities are initially gathered from a wide array of stakeholders, then this initial list is condensed and reduced through guidance of a steering committee, to establish a list of potential research priorities suitable for discussion in an in-person process. In our study, we modified the Delphi approach by having the research team (family medicine clinicians and researchers) conduct the process to combine and condense the initial list, rather than sending out a survey more broadly for prioritization. This was then brought to a group of clinician and researcher stakeholders during an in-person meeting.

We conducted the survey using an online platform (Qualtrics Inc.), optimized for computers as well as mobile devices to ensure ease of use. We solicited responses to the following open-ended query: “*Please suggest up to three important primary care research questions*.” We asked respondents to identify whether they were a “clinician”, “researcher”, or “member of the public”, and asked them to report the country in which they lived. The survey and the study description were available in English, French, and Spanish, and respondents could submit priorities in any of these languages.

Prior to dissemination, the survey was tested and refined with input from stakeholders from the University of Toronto and University of Oxford. Responses were fully anonymous; the survey included a space for participants to submit their email addresses if they were interested in receiving the final results of the study, but these emails were stored separately from the research questions so the origin of the submitted priorities was limited to the demographic information listed above.

The survey was disseminated through a purposive strategy, targeting primarily LMIC stakeholders. We did not limit the survey to LMIC respondents, since preliminary discussions with LMIC colleagues indicated that they felt considering research priorities from high-income countries might be valuable to consider for their own context as well.

We employed a purposive strategy, disseminating the survey through established relationships with primary care/family medicine global health organizations such as the World Organization of National Colleges, Academies and Academic Associations of General Practitioners/Family Physicians (WONCA); past and current participants of the Oxford International Primary Care Leadership Programme; Besrour Centre members (who are family doctors and researchers working in multiple LMIC settings); several national family medicine colleges including members of the College of Family Physicians of Canada and the Royal Australian College of General Practitioners. We also sent out the survey to patient organizations including Patients Canada and the Patient Advisors Network. We encouraged respondents to send the link to colleagues who may be interested. Due to this ‘snowballing’ approach to recruitment, we are unable to identify a denominator of total people who received the survey, in order to calculate a response rate.

The online survey was available from 9 November 2016 to 16 October 2017. During that time, 133 individuals opened the survey; of those, 131 individuals submitted research priorities. These respondents were from 27 countries (S1 Table). On 16 October 2017, the responses were downloaded from Qualtrics into a Microsoft Excel spreadsheet. Responses in French and Spanish were translated into English for data analysis.

All submitted priorities were initially reviewed by two authors (BO and NP). Where there two submitted priorities addressed the same concept, these were combined. Questions that were clearly out of scope were discarded, as were those that were already sufficiently answered based on available evidence. We initially planned to search for supporting evidence for all submitted questions to identify those that should be discarded on the basis that they were already sufficiently answered. We discussed this with a librarian and attempted to search for evidence for 20 randomly selected submitted questions. Given the broad nature of many of the submitted questions, we found that it was very challenging to state whether a particular question was already ‘adequately answered’ such that it should not proceed to the next step in the process, and to do so could inappropriately eliminate important questions, so we did not proceed with conducting detailed searches. We identified six questions for which we were reasonably certain based on our own knowledge, that additional research on that specific topic was unlikely to result in novel information. We have included these questions (as well as all of the questions excluded for any reason) in S3 Table.

Then, all authors (BO, VA, FS, NP, KL, KR) independently ranked each question. This author consensus was used to identify the top 36 submitted priorities.

**Fig 1:**
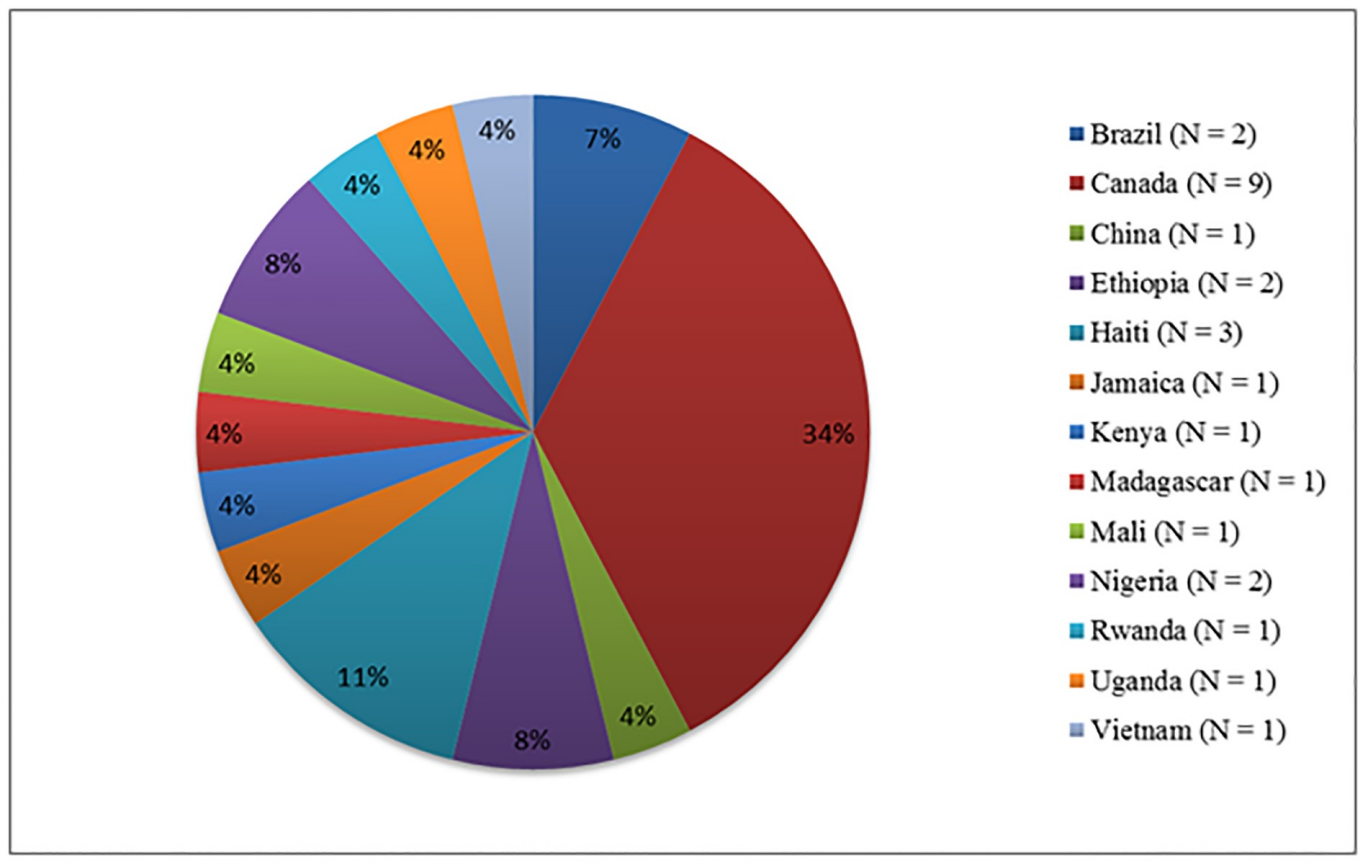
Country of origin of participants at in-person meeting.

The final ranking took place at the Besrour Forum, a yearly international family medicine conference that brings together family medicine clinicians, researchers, and teachers from around the world [21]. We held the meeting during a session at the conference, and all conference attendees took part. All participants provided verbal consent for their involvement. An in-person meeting of 26 participants, representing 13 countries (Fig 1) was held in the afternoon on one of the conference days (8 November 2017). The session was conducted in English. We started the session with one of the authors (BO) describing the purpose of the study, and the process that had been conducted to that point to gather suggested research priorities. We described how we developed the list of the top 36 priorities to be discussed during that session. Participants (the majority of whom were family physicians from LMIC) were then led through an adaptation of the nominal group process to review and rank the top 36 priorities [22], in order to identify the overall top 10. Initially, the group was divided into two equal groups of 13 people, who independently reviewed the top 36 questions, establishing their own ranking of the top 15 questions. Then, the groups came together and discussed to select the top 10.

## Results

There were 379 proposed research priorities submitted in total, by 131 participants from 27 countries (S1 Table). Most were submitted by clinicians (229; 60.4%); the remainder were submitted by members of the public (71; 18.7%) and researchers (79; 20.8%). Most priorities were submitted in the English language (321; 84.7%). 37 were submitted in Spanish (9.8%), and 21 in French (5.5%).

Twenty-nine questions were removed for being ‘out of scope’ (focused on tertiary care issues like the evaluation of surgical technologies, or those focusing on broad epidemiological questions such as worldwide consumption of specific nutrients). A further 65 questions were excluded because the team thought they were very unlikely to end up in the top 36 questions to be reviewed at the final meeting. In general, these were focused on specific contexts or interventions; we assessed that they could be addressed by other more general questions such as those related to electronic communication or health workforce planning. Six questions were excluded because they focused on issues that are already extensively addressed in existing literature (such as prostate cancer screening).

The 279 remaining research priorities were then independently reviewed by two authors (BO and NP) and were combined where possible, resulting in 101 questions for broader review. All authors (BO, VA, FS, NP, KL, KR) independently ranked these 101 questions, resulting in a clear delineation of the top 36 questions based on average rankings.

**Fig 2:**
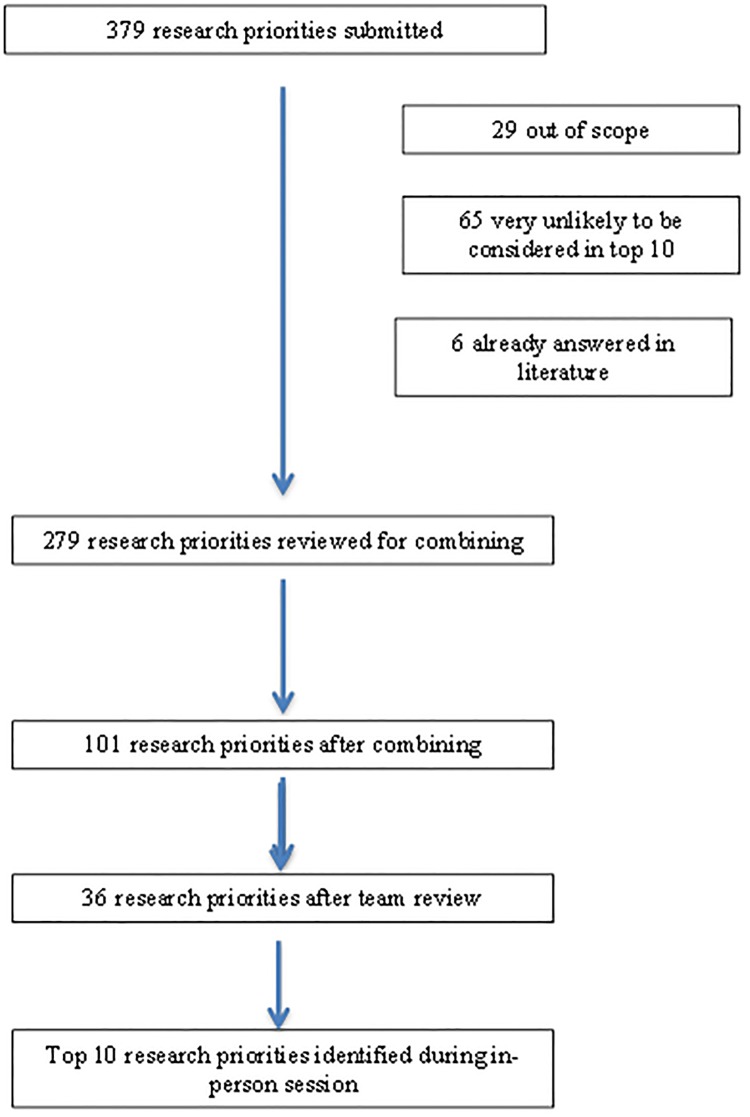
Overview of prioritization process.

An in-person meeting was held during an annual international conference bringing together front line clinicians, teachers, and researchers in family medicine from low- and middle- income countries. It included 26 participants from 13 countries (Fig 1). An overview of the process is shown in Fig 2.

The 10 questions identified during the in-person meeting represented health services issues, specific clinical questions around patient management, and communication between providers (see Table 1). Details of questions that were combined into those identified as the top 10 research priorities are available in supporting information (S2 Table).

**Table 1:** Top 10 unanswered primary care research priorities.

1. How can primary care best address the social determinants of health and promote health equity?
2. How should primary care be financed, organized, and staffed?
3. How best can universal health coverage be achieved in low and middle income countries?
4. How should primary care performance be measured?
5. What are the most effective ways to translate knowledge and evidence into primary care?
6. What are the most effective interventions to improve functional ability and quality of life in people with multimorbidity?
7. What are the best ways to involve patients in the design and delivery of primary care?
8. How can Indigenous communities’ knowledge be integrated into the provision of clinical services at the individual level?
9. How can primary care best promote healthy behaviours in the population?
10. What are the effects of using electronic communication (including email, text messaging and electronic health record access) in the delivery of primary care?

## Discussion

This study was, to our knowledge, the first attempt to identify global research priorities for primary care. It adapted the James Lind Alliance methodology to focus on research priorities for a discipline, rather than a disease-specific area. These priorities represent a diverse set of research areas in fields beyond the reach of previous Primary Care Research [23]. Several of the identified priorities, such as primary care staffing, finance, and workforce issues, are currently areas of inquiry that receive significant attention; their inclusion in the top 10 suggests that more work needs to be done in these areas to strengthen primary care. The focus on universal health coverage is perhaps not surprising, given that this is a key international priority as set out by the World Health Organization [24]. However, progress is clearly lagging in this, and participants from low- and middle- income countries during the in-person meeting identified this as a key barrier to improving the health of communities and countries.

Prioritization efforts have primarily been focused on specific conditions such as post-stroke care [25], eating disorders [26], and Barrett’s oesophagus [14], but have not focused on broader disciplines in order to set out key priorities for those working in a field of inquiry. There has never before been an attempt to identify worldwide primary care research priorities. This is in spite of the fact that high quality primary care has been shown to improve outcomes in highly disparate settings including in low-, middle-, and high- income countries [1,27] but receives comparatively little research funding when compared with investment in basic science [3,28]. Primary care research has been described as the “missing link in the development of high-quality, evidence-based health care for populations” [29]. This mismatch between resources allocated to primary care research and its importance supports the case for research priority setting in this area.

The strengths of this study are the involvement of stakeholders from multiple countries, particularly those who are front-line clinicians in LMIC. We adapted an established, validated process, and were able to conduct an in-person meeting with a highly diverse group of primary care leaders from around the world. Since we were able to combine so many questions from those that were originally submitted, we do not believe that we have disregarded any substantive suggestive research priorities. However, we recognize that study respondents—both those who participated in the online survey and those involved in the in-person meeting—are not necessarily representative of all stakeholders in “international” primary care. It would be impossible to establish a group that is ‘representative’ of international primary care stakeholders; we attempted to engage as diverse as possible a group through purposive sampling to obtain submissions. The final list of research priorities therefore cannot necessarily be suggested as representative of all international primary care stakeholders. A different group of stakeholders, and a different research team, may have identified a different list of key priorities. We believe the diversity of respondents has ensured the top 10 list includes important priorities that are valued in a variety of contexts, including low-, middle-, and high-income countries. Another key limitation was relatively limited participation by patients and members of the public (only 18.7% of total priorities were submitted by this group); this is similar to the rate of participation from some JLA priority setting partnerships in the literature [30] but considerably less than others [14,25]. It is not known how this affected the results.

In conclusion, we facilitated a priority setting process to identify the top 10 global primary care research priorities. This activity compliments other efforts to promote primary care research and to establish new directions for it, particularly in LMIC, such as the PHPCI [11]. These priorities will be useful for researchers, for policymakers, and for research funding organizations, to understand what might be the most important priorities to address through research in primary care and family medicine, in order to improve health for all.

## Supporting information

S1 Table List of countries of origin of submitted research priorities. (DOCX)

S2 Table Submitted research priorities combined into those rated as ‘top 10’. (DOCX)

S3 Table Excluded questions. (DOCX)

## References

[1] Starfield B, Shi L, Macinko J. Contribution of primary care to health systems and health. Milbank Q. 2005;83(3):457–502. 10.1111/j.1468-0009.2005.00409.x 16202000 PMC2690145

[2] Rohde J, Cousens S, Chopra M, et al 30 years after Alma-Ata: has primary health care worked in countries? Lancet. 2008;372(9642):950–61. 10.1016/S0140-6736(08)61405-1 18790318

[3] Mant D, Del Mar C, Glasziou P, Knottnerus A, Wallace P, van Weel C. The state of primary-care research. Lancet. 2004;364(9438):1004–6. 10.1016/S0140-6736(04)17027-X 15364194

[4] Brown LJ, McIntyre EL. The contribution of Primary Health Care Research, Evaluation and Development-supported research to primary health care policy and practice. Aus J Public Health. 2014;20(1):47–55. 10.1071/PY12058 23092638

[5] De Maeseneer J, van Weel C, Egilman D, Mfenyana K, Kaufman A, Sewankambo N. Strengthening primary care: addressing the disparity between vertical and horizontal investment. Br J Gen Pract. 2008;58(546):3–4. 10.3399/bjgp08X263721 18186987 PMC2148229

[6] Fleurence R, Whicher D, Dunham K, Gerson J, Newhouse R, Luce B. The Patient-centered Outcomes Research Institute’s Role in Advancing Methods for Patient-centered Outcomes Research. Medical Care. 2015;53(1):2–8. 10.1097/MLR.0000000000000244 25334055 PMC4336314

[7] Pickler RH, Tubbs-Cooley HL. Patient-centered outcomes research: a "new" research agenda. J Pediatric Health Care. 2014;28(1):101–4. 10.1016/j.pedhc.2013.08.004 24100007

[8] Borden WB, Chiang YP, Kronick R. Bringing Patient-Centered Outcomes Research to Life. Value Health. 2015;18(4):355–7. 10.1016/j.jval.2015.01.010 26091588

[9] Gold R, Whitlock EP, Patnode CD, McGinnis PS, Buckley DI, Morris C. Prioritizing research needs based on a systematic evidence review: a pilot process for engaging stakeholders. Health Expect. 2013;16(4):338–50. 10.1111/j.1369-7625.2011.00716.x 21838830 PMC3218292

[10] Mant D. National Working Group Report. R & D in Primary Care. London: Department of Health, 1997.

[11] Bitton A, Ratcliffe HL, Veillard JH, et al Primary Health Care as a Foundation for Strengthening Health Systems in Low- and Middle-Income Countries. J Gen Intern Med. 2017;32(5):566–71. 10.1007/s11606-016-3898-5 27943038 PMC5400754

[12] Chalmers I, Glasziou P. Avoidable waste in the production and reporting of research evidence. Lancet. 2009;374(9683):86–9. 10.1016/S0140-6736(09)60329-9 19525005

[13] Cowan K, Oliver S. The James Lind Alliance Guidebook, Version 5 [Internet]. London, UK: The James Lind Alliance; 2013 [cited 2018 February 12]. http://www.jlaguidebook.org/pdfguidebook/guidebook.pdf

[14] Britton J, Gadeke L, Lovat L, et al Research priority setting in Barrett’s oesophagus and gastro-oesophageal reflux disease. Lancet Gastroenterol Hepatol. 2017; 2(11): 824–31. 10.1016/S2468-1253(17)30250-9 28867477

[15] De Maeseneer JD, Weel Cv, Egilman D, et al Funding for primary health care in developing countries. BMJ. 2008;336(7643):518 10.1136/bmj.39496.444271.80 18325939 PMC2265338

[16] Van Royen P, Beyer M, Chevallier P, et al The research agenda for general practice/family medicine and primary health care in Europe. Part 3. Results: person centred care, comprehensive and holistic approach. Eur J Gen Pract. 2010;16(2):113–9. 10.3109/13814788.2010.481018 20438283

[17] Stoffers J. Research priorities in family medicine. Eur J Gen Pract. 2011;17(1):1–2. 10.3109/13814788.2011.555617 21344983

[18] Schafer W, Groenewegen PP, Hansen J, Black N. Priorities for health services research in primary care. Qual Prim Care. 2011;19(2):77–83. 21575330

[19] Hasson F, Keeney S, McKenna H. Research guidelines for the Delphi survey technique. J Adv Nurs. 2000;32(4):1008–15. 11095242

[20] De Villiers MR, De Villiers PJ, Kent AP. The Delphi technique in health sciences education research. Med Teacher. 2005;27(7):639–43. 10.1080/13611260500069947 16332558

[21] Besrour Centre. The Besrour Forum [Internet]. Mississauga, ON: College of Family Physicians of Canada; 2017 [cited 2018 March 22]. https://fmf.cfpc.ca/besrour-forum/.

[22] Gill PJ, Hewitson P, Peile E, Harnden A. Prioritizing areas for quality marker development in children in UK general practice: extending the use of the nominal group technique. Fam Pract. 2012;29(5):567–75. 10.1093/fampra/cms006 22308179

[23] Kidd M, Heath I, Howe A, eds. Family Medicine: The Classic Papers. Boca Raton: CRC Press; 2016.

[24] Tokyo Declaration on Universal Health Coverage: All Together to Accelerate Progress Towards UHC [Internet]. Geneva: World Health Organization; 2017 [cited 2018 March 22]. http://www.who.int/universal_health_coverage/tokyo-decleration-uhc.pdf?ua=1.

[25] Pollock A, St George B, Fenton M, Firkins L. Top 10 research priorities relating to life after stroke—consensus from stroke survivors, caregivers, and health professionals. Int J Stroke. 2014;9(3):313–20. 10.1111/j.1747-4949.2012.00942.x 23227818

[26] van Furth EF, van der Meer A, Cowan K. Top 10 research priorities for eating disorders. Lancet Psych. 2016;3(8):706–7. 10.1016/S2215-0366(16)30147-X 27475763

[27] Macinko J, Starfield B, Erinosho T. The impact of primary healthcare on population health in low- and middle-income countries. J Amb Care Manag. 2009;32(2):150–71. 10.1097/JAC.0b013e3181994221 19305227

[28] Beasley JW, Starfield B, van Weel C, Rosser WW, Haq CL. Global health and primary care research. J Am Board Fam Med. 2007;20(6):518–26. 10.3122/jabfm.2007.06.070172 17954858

[29] Rosser WW, van Weel C. Research in family/general practice is essential for improving health globally. Ann Fam Med. 2004;Suppl2:S2–4. 10.1370/afm.145 PMC1466769 15655083

[30] Reay H, Arulkumaran N, Brett SJ. Priorities for Future Intensive Care Research in the UK: Results of a James Lind Alliance Priority Setting Partnership. J Intensive Care Soc. 2014;15(4):288–96. 10.1177/1751143715609954 PMC5606390 28979474

