# Supplementary material for: Identifying top 10 primary care research priorities from international stakeholders using a modified Delphi method
Source: PLoS One. 2018 Oct 25;13(10):e0206096. doi: 10.1371/journal.pone.0206096 (PMC6201922; doi:10.1371/journal.pone.0206096)
Supplement: S1 Table — (DOCX) [file pone.0206096.s001.docx]

**Supporting information S1.** List of countries of origin of submitted research priorities

| **Country** | **Number of priorities submitted** | **Number of respondents** | **Low/middle/high income?*** |
| --- | --- | --- | --- |
| Argentina | 5 | 2 | High |
| Australia | 108 | 36 | High |
| Bahamas | 3 | 1 | High |
| Belgium | 2 | 1 | High |
| Brazil | 7 | 3 | Middle |
| Canada | 97 | 32 | High |
| Denmark | 2 | 1 | High |
| Equador | 3 | 1 | Middle |
| Spain | 19 | 7 | High |
| Haiti | 3 | 1 | Low |
| Indonesia | 3 | 1 | Middle |
| Ireland | 3 | 1 | High |
| Kenya | 4 | 2 | Middle |
| Madagascar | 3 | 1 | Low |
| Malawi | 11 | 4 | Low |
| Mali | 6 | 2 | Low |
| Netherlands | 6 | 2 | High |
| Nigeria | 9 | 3 | Middle |
| Norway | 2 | 1 | High |
| Rwanda | 3 | 1 | Low |
| Trinidad and Tobago | 6 | 2 | High |
| Tunisia | 3 | 1 | Middle |
| Turkey | 6 | 2 | Middle |
| Uganda | 3 | 1 | Low |
| United Kingdom | 16 | 6 | High |
| United States of America | 42 | 14 | High |
| Venezuela | 4 | 2 | Middle |
| *Total* | 379 | 131 |  |

*As classified by the World Bank: https://datahelpdesk.worldbank.org/knowledgebase/articles/906519-world-bank-country-and-lending-groups
