# Supplementary material for: Identifying top 10 primary care research priorities from international stakeholders using a modified Delphi method
Source: PLoS One. 2018 Oct 25;13(10):e0206096. doi: 10.1371/journal.pone.0206096 (PMC6201922; doi:10.1371/journal.pone.0206096)
Supplement: S2 Table — (DOCX) [file pone.0206096.s002.docx]

**Supporting Information S2.** Submitted research priorities combined into those rated as ‘top 10’

1. How can primary care best address the social determinants of health and promote health equity?
   1. What information or data points related to social determinants of health would be most actionable for primary care practices?
   2. How can primary care providers effectively assess and manage patient care issues related to the social determinants of health?
   3. What can frontline health workers do to take action on social determinants of health?
   4. How can primary health care better meet the needs of the most marginalized and disadvantaged groups?
   5. What factors impact the choice of family medicine residents to pursue work with marginalized and underserved groups?
   6. How to reverse the inverse care law?
   7. What social interventions (such as providing income, housing, transportation) are most effective at reducing functional impairment and death amongst patients in primary care?
   8. How can primary care address the social determinants of health?
   9. How can the "inverse care law" best be mitigated by primary care?
   10. How can we improve compliance in communities with poverty and low health literacy?
   11. What strategies will support GPs to manage low socioeconomic patients with severe chronic mental health problems in order to optimize their health?
   12. How do we begin to develop food security?
   13. How do we address the question of homelessness and unsafe housing?
   14. How can we interest disadvantaged patients in preventive health care?
2. How should primary care be financed, organized, and staffed?
   1. What is the optimal panel size to deliver patient focused, value added healthcare?
   2. What are the strategies to ensure enough family doctors for everyone, with same-day or next-day access for all their patients facing illness?
   3. What is the most effective way to provide care to rural and other underserved populations?
   4. How to improve timely access to care?
   5. What should be the components of primary care?
   6. What is (should be) the role of Family Physicians in PHC in LMICs
   7. Which primary care models are effective in low-resource settings like Malawi?
   8. What is the optimal composition of primary care teams in different contexts?
   9. What are the roles of family medicine physicians in LMIC?
   10. What practice model will allow for improved access to PHC services given the current workforce?
   11. How can primary care systems integrate with public health systems?
   12. How can we curb 'doctor shopping'?
   13. Do full-time doctors provide better care by virtue of greater availabilty and therefore continuity than part-time ones?
   14. Do various funding models make a difference to patient care?
   15. Is corporate ownership of GP practices better than doctor owned?
   16. Do longer consultations = improved health outcomes +/- lower health costs?
   17. Services that patient would like to access from Family Physician/ Practice
   18. What is the best model for Afterhours care of patients who are not emergencies?
   19. Should GPs be required to be contactable by police, hospital, pathology etc when they are not on duty or call?
   20. Are multidisciplinary teams in chronic care more or less cost efficient than general practice care alone?
   21. For developed countries do family physicians or interprofessional specialty clinics (e.g. diabetes clinics with specialists and nurses or nurse practitioners) provide the most cost-efficient and effective care for major disease groups such as diabetes, heart failure, stroke?
3. How best can universal health coverage be achieved in low and middle income countries?
   1. What interventions will ensure universal coverage with basic primary care packages in middle and low income countries?
   2. What are the most important barriers to implement primary care?
   3. How can the pivotal role of family medicine in high-quality cost-effective healthcare be explained to policymakers and community members?
   4. What are the most effective strategies for promotion of and advocacy for family medicine as the best model of care in low, middle and high income countries?
   5. What factors influence governments to invest in primary health care sector, and specifically family medicine?
   6. What role can a primary care doctor play in a developing country, given the prevailing war between public health and family medicine for finances and empowerment in those countries?
   7. What are the key factors that influence the desirability of consulting a family physician in low and middle income countries
   8. What are the best strategies to strengthen PHC as a strategy for health delivery to the community?
   9. Strategies to achieve the maximum benefit to the patient with the best rational use of resources
   10. How to make visible and give value to the care offered primary care?
   11. How to convince stakeholders (patients, clinicians, governments) the benefits of continuity of care
   12. What is the best way to ensure the delivery of quality care to people of refugee background and other culturally and linguistically diverse communities?
   13. What public policy changes will encourage more medical students to go into primary care medicine?
   14. What are the most effective interventions to recruit and sustain Family Physicians to rural communities in low, middle, and high income countries
   15. What are the main satisfaction areas of family physicians that they promote in order to suggest a career in family medicine to medical students?
   16. What is the perception, knowledge and attitudes for non-family medicine specialists over family medicine specialists?
   17. What makes people respect family medicine, and how can you convey this where there is not yet a robust family medicine model?
   18. What are the most effective interventions to recruit family doctors to and maintain them in rural communities?
   19. What are the best strategies to make undergrad students choose Family Medicine as a career?
   20. What are the most effective interventions to recruit family physicians to rural communities in low, middle, and high income countries?
   21. How can we recruit and retain excellent doctors and allied health professionals?
   22. What is the most effective strategy to get family physicians to work in primary health care centres in developing countries?
   23. What factors impact the choice of medical students to enter primary care in low, middle, and high income countries?
   24. How to increase interest in Family Medicine among medical students and other stakeholders in LMICs?
   25. What are the most effective interventions to promote the development of human resources in family medicine?
   26. What measures have governments taken to encourage doctors to work in rural areas among the experiences of low-income countries?
   27. How can we optimise GP training?
   28. What are the effects of private health companies providing PHC in LMIC in terms of quality of care (access to health care, continuity and comprehensive care) and in terms of its socioeconomic impact (jobs created, income and income inequality generated, social inequality)? Will these initiatives provide a better health care or will they worsen the inverse care law?
   29. What is the influence of industry on the current over investigation and treatment of blood lipids in primary care?
   30. Is the cost of primary care becoming out of the reach of many families?

4. How should primary care performance be measured?

1. How can primary care be measured and strengthened in low income countries?
2. How feasible is it to collect the data for proposed primary health care measures (including workforce), and how robust are they?
3. How can we measure team function in primary care?
4. How to measure the impact of Family Medicine on primary care health service delivery?
5. How to measure the impact of family physicians on improving the health of the populations served?
6. What would be the impact of implementing a quality approach on improving the quality of care in a family medicine setting?
7. What is effective primary care?
8. How do we distinguish between demand and need?
9. What is an acceptable level of access to primary care?
10. How do we accurately measure GP workload?
11. What is the most efficient method to detect inappropriate investigations (primarily pathology testing) ordered by clinicians?

5. What are the most effective ways to translate knowledge and evidence into primary care?

1. How can we create a worldwide learning community to promote improvements in primary care delivery?
2. Do primary care settings that implement evidence based care actually have better patient outcomes?
3. What is the most effective way to provide evidence based research to busy primary care practitioners?
4. What strategies are available to assist with interpretation of new medical information?
5. How should family physicians best translate findings from high quality studies to the patients they care for that would have been likely excluded from the studies due to multiple end stage co-morbidities?
6. What are the most effective interventions to disseminate knowledge to keep continuing medical education for personal growth?
7. What is the best way to train/provide on-going support for front-line health workers in low resource settings?
8. What is the best way to implement integrated evidence-based health packages in low resource settings?
9. What are the best methods for implementing evidence-based practices in primary care?
10. What are the most effective interventions to transfer the best evidence to clinical practice?
11. How do we improve the quality and safety of primary care provision?
12. How can evidence inform quality primary care provision and how do we make it sustainable?
13. How best to prioritize daily questions in general practice through pragmatic randomised clinical trials?
14. Does GP knowledge of and practice of evidence based medicine principles improve patient clinical outcomes?
15. What is the most effective system to collate and disseminate local disease prevalence and incidence data?

6. What are the most effective interventions to improve functional ability and quality of life in people with multimorbidity?

1. What are the most effective interventions in the comprehensive risk management of non-communicable diseases in primary care?
2. What is the influence of substance use on self-management of chronic disease?
3. How to improve prevention and management of chronic conditions?
4. What is the influence of the quality of the doctor-patient communication on patient outcomes in chronic diseases in primary care
5. What is the effect of the quality of the doctor-patient relationship on patient outcomes in chronic diseases in primary care
6. What is the effectiveness of continuity of care on patient outcomes in chronic diseases in primary care
7. How to explore the impact of Family Medicine on morbidity and mortality among patients with non-communicable diseases?
8. What is the best way to standardize care for patients with more than one condition and do not match patients eligible for guidelines limited to an organ or disease?
9. How to treat patients with multimorbidity?
10. What strategies to improve adherence to treatment of patients with chronic diseases such as Diabetes and Hypertension?
11. What are the most effective interventions to improve functional ability in people with multimorbidity?
12. What are the most effective interventions to improve quality of life in people with multimorbidity?
13. How to assess the benefits of multiple medications for multiple conditions?
14. What is the most appropriate way to deprescribe in multi morbidity?
15. How should multi-morbidity be addressed in clinical guidelines?

7. What are the best ways to involve patients in the design and delivery of primary care?

1. How can patients partner with primary care to be part of the team, such as having access to EMR for example?
2. What factors make it possible (easier) for patients' priorities to be recognized and addressed in primary healthcare settings?
3. What are the most effective methods to improve patient engagement in their healthcare?
4. What kinds of incentives or payment models would support greater shared decision-making in primary care practices?
5. Does the use of off-line, localized, integrated clinical decision support resources by diagnostic decision makers serving within a Kenyan health system influence patient care outcomes?
6. What is the value of the active and voluntary participation of the community in setting up an equitable first line primary care system?
7. How do we involve patients in our research projects right from the start?
8. What are the determining factors that favour community participation in decision making?
9. What are the most effective interventions in the training of patients to be responsible for their health care?
10. What are the best ways to involve patients in their own care?
11. How do we incorporate patient reported outcome measures into health policy
12. What are the treatment outcomes that patients would like to experience from their Family Physician/Practice?
13. What is the optimal person-centred approach to improve patients' satisfaction, knowledge and quality of life?
14. What are the most effective strategies to build primary care provider readiness and support to engage patients & families in practice improvement work?

8. How can Aboriginal communities’ knowledge be integrated into the provision of clinical services at the individual level?

9. How can primary care best promote healthy behaviours in the population?

1. What techniques can be used by primary care practitioners to effectively encourage patients to avoid obesity?
2. How do we help patients follow doctor instructions to improve their heath and avoid costly surgery or other outcomes?
3. What is the most effective action I can take to stay healthy?
4. What is the role of preventative medicine in primary health care?
5. How can we promote more supportive environments for health at community and national levels?
6. What are the most effective ways to help lifestyle behaviour change in a consultation?
7. Which weight loss strategies would be most effective for persons living in the Caribbean?
8. How can primary care most effectively engage with local population-level prevention strategies?
9. How to effectively promote lifestyle changes to make people healthier?
10. How best to address childhood obesity?
11. What government policy works to promote healthy eating in remote or poor communities?
12. How to we improve adherence to lifestyle measures in primary care?
13. What are effective strategies to improve diet?
14. What are effective strategies to improve diet in remote Australian communities?
15. How can we best convey to the public and to our patients that most of the time it is how they care for themselves that makes more difference to their health than anything that we or other parts of the health care system might do for them?
16. Are young people able to access the required primary care that they need to enhance preventive health practices?
17. What are the enablers for choosing healthy lifestyle habits?
18. What interventions work to prevent obesity in children, adolescents and adults?

10. What are the effects of using electronic communication (including email, text messaging and electronic health record access) in the delivery of primary care?

1. What is the best way to use primary care electronic medical record data to evaluate population health interventions?
2. What are the top patient priorities in primary care, for example: remote appointments, email, ease of access?
3. It can be unsafe for elderly rural patients to drive to cities for specialist appointments. Could a way be found to bring virtual care appointments to their small town primary care provider offices?
4. Can primary care be effectively delivered through electronic means (e.g. Facetime, etc.)?
5. How can we use technology to change the patient/doctor relationship for the benefit of both?
6. How can primary care physicians be supported to be the centre of care and team lead for their patient's various specialists and hospitalizations, etc.?
7. Are patient outcomes improved and readmissions avoided when family/care partners are actively involved in care planning and transitions?
8. How to speed up medical records communications between disconnected providers, especially in the case of acute patient needs, to ensure continuity of care?
9. What are the optimal ways for physicians to interact both with electronic medical records on computers and patients in the same room?
10. What is the possibility of a secure physician website information regarding controlled drugs prescribed to a specific person?
11. What is the most effective means of communicating with general practitioners?
