# Supplementary material for: Identifying top 10 primary care research priorities from international stakeholders using a modified Delphi method
Source: PLoS One. 2018 Oct 25;13(10):e0206096. doi: 10.1371/journal.pone.0206096 (PMC6201922; doi:10.1371/journal.pone.0206096)
Supplement: S3 Table — (DOCX) [file pone.0206096.s003.docx]

**Supporting Information S3.** Excluded questions

Authors’ note: Some of the questions below have been edited for readability.

Questions excluded because they were assessed as already being adequately answered by existing research

| Would prescriptions for exercise, with interval follow up appointments improve population health outcomes? |
| --- |
| Is there any benefit to an annual medical evaluation? |
| What is the optimal frequency of visits for a preventative health check in a healthy adult under age 65? |
| Should the PSA test +/- DRE be used to screen for prostate cancer? |
| What are the most effective ways to treat chronic non-cancer pain? |
| How can we manage infections in this era of high antibiotic resistance? eg UTI, skin infections |

Questions excluded for being ranked by the research team as being ‘very unlikely to end up in the top 15’

| In-home assistance with personal care can be necessary but an affront to elderly patients. Are there innovative ways to bathe with help that would be more acceptable? |
| --- |
| Elderly couples do not necessarily need the same level of care. How could current models of residential care be adapted to provide multiple levels of care (e.g. nursing care, independent living) under the same roof so couples could stay together? |
| What is the best way for patients to organize medical documents? |
| What is the proper procedure for washing of hands and what type of soap to minimize the spread of diseases such as community and hospital acquired c-dif etc.? |
| Are there more natural means by which antibiotic resistant deseases such as MRSA can be brought under control or cured as opposed to throwing more antibiotics at the issue? |
| Do hospitalists improve care outcomes? |
| What can be done to improve upon timely communication between doctors? |
| How can discover a way to have affordable home care for a loved one who is ill, and I am working? |
| How can we ensure that families with chronically ill children are adequately monitored and treated while at home in the community? |
| How many families of chronically ill patients deal with caregiver burnout and try to avoid creating their own medical issues? |
| How can we measure the disparity of services available to patients between in-hospital and in the community? |
| How do we assist patients who must navigate multiple health systems for to seek health care (especially systems which are not connected via EMR, such as different competing systems, behavior health providers, complimentary medicine providers)? How to ease the burden on patients/families who currently must do all the communication between providers and/or make multiple contacts to Medical Records to attempt to help providers communicate? |
| In times of disease outbreaks, such as measles, how to keep primary care clinic environments safe for vulnerable/un-immunized patients? |
| How much the leaders understand primary care in each country? |
| How do family physicians describe their professional identity and why? |
| What approaches should physicians utilize to ensure that the information gathering portion of a patient encounter becomes, in itself, a therapeutic intervention? |
| How is an activated patient identified in clinical practice? |
| How are opioid addicted patients identified and managed in primary care? |
| What are the top 3 issue(s) you discuss with your family doctor on an annual basis? |
| How to implement CQI activities as part of the routine practice? |
| What is the knowledge/education level of patients with chronic diseases (HTA, Diabetes)? |
| What are the most effective interventions of a Family Physician to community interventions in low resource setting (home visit) ? |
| What percentage of clinicians in low income countries know quality improvement concept? |
| What are the mostly seen problems of immigrants in primary care? |
| Are family physicians feel themselves competent for cultural issues? |
| How can we achieve appropriate use of antibiotics for non-specific symptoms in a nursing home setting? |
| How can resident/physician exchange programs be organized to improve exposure and care in rural populations? |
| What is the best way to control the intake of anti-tuberculosis drug of TB patients in rural area? |
| How to create a good family support in elderly cancer patients? |
| How optimal is primary care where I practice (Trinidad, Caribbean) |
| Is screening mammography cost effective? |
| How can effective interprofessional collaboration be promoted in research projects? |
| Efficacy and efficiency of alternative medicine vs the traditional in the rural environment of low resources |
| How can family doctors influence health policies in their countries? |
| Why is the family doctor still considered a general practitioner? |
| Is influenza vaccination effective in diabetics? |
| Is influenza vaccination effective in people over 65 years of age? |
| What are the expectations of patients attending a primary care visit? |
| What are the beliefs of chronic patients not to take the medication? |
| How can primary care promote adaptation to climate change? |
| How can primary care reduce motor vehicle use? |
| What is the most effective way to improve access to long acting reversible contraception? |
| What prophylaxis is recommended for long haul flights following previous provoked below knee DVTs? |
| Which non-barrier method contraception is best for patients with a history of depression or anxiety? |
| Who needs antibiotic therapy for URTI? |
| Who needs a statin for primary prevention? |
| Does medical acupuncture work better than GP care for back pain? |
| Does medical acupuncture work better than GP care for headache? |
| Does medical acupuncture work better than GP care for neck pain? |
| Testosterone replacement in men and women as we age? |
| Does a low sugar/low gluten/low CHO etc diet improve mood +/- health outcomes |
| What is the next best oral hypoglycaemic to use after metformin for aiding weight loss with improved glycemic control? |
| How long to continue with diet and exercise in the morbidly obese before considering bariatric surgery? |
| How well do patients understand their cancer treatment benefits/ risks? |
| At what level should blood pressure and cholesterol be treated? |
| Is physiotherapy/exercise effective for back pain, hip pain, shoulder pain? |
| Are there effective treatments for the pain of back pain, hip pain, etc? |
| What is an effective, implementable, non-surgical solution for obesity in Australia? |
| What language is best when de-medicalising low grade depression and anxiety? |
| Why are 1st world govts more happy supporting nurse-proceduralists than GP-proceduralists? |
| What is the best medication to assist patients reduce alcohol use, in the absence of the desire to abstain? |
| How can we reduce bullying within the medical profession? |
| Does keeping people in detention affect their mental health and their ability to later assimilate into their new communities? |
| What is the effectiveness of a community health approach in drug addiction programmes? |
| Can the patient add information to his files at the general practice? |

Questions excluded because they were assessed as ‘out of scope’

| What is the optimal turnaround time from ER to Hospital Admission? |
| --- |
| How can a Hospitalist & Patient relationship be improved upon? |
| How can Primary Care better connect/educate patients and themselves about research options as part of a pt's treatment plan? |
| How to find additional help from the community? |
| How to find the best volunteer in the community? |
| When does a PCP send their patient to a specialist? |
| How are the best practices in delivering coordinated care across healthcare delivery systems? |
| What are the characteristics of an effective care management program? |
| passion for community care-it's not about $$$ |
| What are optimal interventions for promoting self-efficacy in chronic care management? |
| What is the role of prevention at primary care? |
| Community based medical education spiral curiculum in medical training |
| How can we redress the balance of prevention to curative work? |
| What are the priority areas of research in family medicine in developing countries? |
| What are the screening tests that should be recommended? |
| What is the value of permanent connections to a control center? For example, defibrillators. |
| What are the most common health risks in the first line? How to investigate and prevent them? |
| Distance education programs to make compatible the continuous medical education with the assistance activity |
| How to improve the promotion of health in the population if they are rooted in curative medicine? |
| How to increase the resolving capacity of primary care professionals? |
| What is the value of anamnesis and physical examination in the diagnosis of acute and chronic diseases in primary care? |
| Dietary factors in world health and research into ways of improving this either as a result of excess or deprivation |
| Dietary salt intake world wide |
| World wide incidence of diseases and contributing factors |
| Governments could consider introducing access to free gym facilities, which could people working extra 7 yrs, and could double their retirement funds on stock market over 7 yrs and solves pressure on aged pensions |
| How to get the public to know that GP is a speciality? |
| Why does research get repeated without research to see why not implemented last time? |
| When does a GP give antibiotics when there is sinusitis and when not? |
| Does the GP makes ECG's [electrocardiograms] and is he able to assess them? |
